# Epithelial-Stromal Interactions in Human Breast Cancer: Effects on Adhesion, Plasma Membrane Fluidity and Migration Speed and Directness

**DOI:** 10.1371/journal.pone.0050804

**Published:** 2012-12-10

**Authors:** Cristiana Angelucci, Giuseppe Maulucci, Gina Lama, Gabriella Proietti, Anna Colabianchi, Massimiliano Papi, Alessandro Maiorana, Marco De Spirito, Alessandra Micera, Omar Bijorn Balzamino, Alba Di Leone, Riccardo Masetti, Gigliola Sica

**Affiliations:** 1 Istituto di Istologia ed Embriologia, Università Cattolica del Sacro Cuore, Roma, Italia; 2 Istituto di Fisica, Università Cattolica del Sacro Cuore, Roma, Italia; 3 Istituto di Ricovero e Cura a Carattere Scientifico - Fondazione G.B. Bietti, Roma, Italia; 4 Dipartimento per la Tutela della Salute della Donna e della Vita Nascente, del Bambino e dell'Adolescente - Unità Operativa di Chirurgia Senologica, Facoltà di Medicina e Chirurgia “A. Gemelli”, Università Cattolica del Sacro Cuore, Roma, Italia; University of Edinburgh, United Kingdom

## Abstract

Interactions occurring between malignant cells and the stromal microenvironment heavily influence tumor progression. We investigated whether this cross-talk affects some molecular and functional aspects specifically correlated with the invasive phenotype of breast tumor cells (i.e. adhesion molecule expression, membrane fluidity, migration) by co-culturing mammary cancer cells exhibiting different degrees of metastatic potential (MDA-MB-231>MCF-7) with fibroblasts isolated from breast healthy skin (normal fibroblasts, NFs) or from breast tumor stroma (cancer-associated fibroblasts, CAFs) in 2D or 3D (nodules) cultures. Confocal immunofluorescence analysis of the epithelial adhesion molecule E-cadherin on frozen nodule sections demonstrated that NFs and CAFs, respectively, induced or inhibited its expression in MCF-7 cells. An increase in the mesenchymal adhesion protein N-cadherin was observed in CAFs, but not in NFs, as a result of the interaction with both kinds of cancer cells. CAFs, in turn, promoted N-cadherin up-regulation in MDA-MB-231 cells and its *de novo* expression in MCF-7 cells. Beyond promotion of “cadherin switching”, another sign of the CAF-triggered epithelial-mesenchymal transition (EMT) was the induction of vimentin expression in MCF-7 cells. Plasma membrane labeling of monolayer cultures with the fluorescent probe Laurdan showed an enhancement of the membrane fluidity in cancer cells co-cultured with NFs or CAFs. An increase in lipid packing density of fibroblast membranes was promoted by MCF-7 cells. Time-lapsed cell tracking analysis of mammary cancer cells co-cultured with NFs or CAFs revealed an enhancement of tumor cell migration velocity, even with a marked increase in the directness induced by CAFs.

Our results demonstrate a reciprocal influence of mammary cancer and fibroblasts on various adhesiveness/invasiveness features. Notably, CAFs' ability to promote EMT, reduction of cell adhesion, increase in membrane fluidity, and migration velocity and directness in mammary cancer cells can be viewed as an overall progression- and invasion-promoting effect.

## Introduction

The leading role of epithelial-stromal dialogue in the development of the mammary gland has been well recognized, but accumulating evidence has demonstrated that in breast cancer altered interactions occurring between epithelial malignant cells and the associated fibroblasts play a major part in tumor development, growth and progression [1]–[4]. The resulting transformed microenvironment, also-called reactive stroma, differs from the stroma of the healthy mammary gland, showing disruptions in the fibroblast-epithelial cell cross-talk in terms of cell proliferation and extracellular matrix remodelling [5], [6]. In particular, the migratory/invasive behaviour of tumor cells seems to be strongly influenced by this aberrant dialogue with the adjacent fibroblasts [5], [7]. The release of soluble factors by both kinds of cell types reciprocally influences their peculiar properties, creating suitable conditions for malignant cells not only to multiply but also to migrate and invade other tissues, outside the boundaries of the mammary gland [2], [3], [8]–[12]. Fibroblasts arising from tumor stroma, the so-called cancer-associated fibroblasts (CAFs), compared to normal fibroblasts (NFs), have acquired distinct properties mainly leading to the promotion of cancer cell proliferation and invasion. Differences in the activity of NFs versus CAFs in breast tumors may result from alterations in molecular and/or cellular mechanisms that are responsible for the production and release by CAFs of a number of soluble factors such as fibroblast growth factors [13], transforming growth factor-β (TGF-β) [14], insulin-like growth factors [15], and hepatocyte growth factor (HGF) [12]. Tumorigenicity of CAFs, derived from breast tumors and injected together with malignant cells, has been widely demonstrated in animal models [8], [16], [17]. Induction of mammary cancers has also been demonstrated in mice orthotopically grafted with TGF-β-and/or HGF-transfected fibroblasts co-injected with apparently normal epithelial breast cells, highlighting the critical role of heterotypic interactions in human breast development [17]. This tumor-stroma cross-talk seems to have an important influence also in the involved lymph-node microenvironments, as demonstrated by the ability of nodal fibroblasts to affect viability, proliferation and migration of breast cancer cells [18]–[20]. At the root of the alterations in these latter activities seems to be the induction of reciprocal changes in the genomic profiles of cancer and stromal cells involving, in particular, genes critical for growth control, cell adhesion and invasiveness [19]–[22].

Despite the growing number of studies focused on epithelial-stromal interactions in solid tumors, the role played by fibroblasts in the development and progression of breast cancer is not yet fully understood. Thus, the use of relevant co-culture models using fibroblasts derived from normal and malignant stroma may provide a practical tool for the analysis of reciprocal influences between the stroma and the epithelial tumor compartment.

In the present report, we sought to gain a deeper insight into some molecular and functional properties strongly correlated with cancer progression and metastatization. These properties may be supposed to be reciprocally influenced in the epithelium-stroma cross-talk occurring in breast cancer. With this aim, we performed breast cancer cells/fibroblasts co-culture assays and analyzed changes occurring in adhesion molecule expression, membrane fluidity, and migration velocity and directness in both the cell types. In our experimental approach, we co-cultured estrogen receptor (ER)-positive, poorly invasive and low metastasizing mammary cancer cells (MCF-7) or ER-negative, highly invasive and metastatic breast cancer cells (MDA-MB-231) with fibroblasts isolated from mammary healthy skin (NFs) or from breast tumor stroma (CAFs) in conventional 2D adherent cultures or in a 3D system (nodules). Results obtained were compared with those from the respective homotypic cultures.

## Materials and Methods

### Breast cancer cell lines

The estrogen receptor (ER)-positive, poorly invasive, and low metastasizing human breast carcinoma cell line MCF-7 and the ER-negative, highly invasive, and metastatic human breast carcinoma cell line MDA-MB-231 were obtained from American Type Culture Collection (Manassas, VA, USA). MCF-7 and MDA-MB-231 cells were maintained in Dulbecco's Modified Eagle's Medium (DMEM, Euroclone, Milan, Italy) supplemented with 10% fetal bovine serum (FBS, Life Technologies, Paisley, Scotland), antibiotics (100 IU/ml penicillin, 100 µg/ml streptomycin, Euroclone) and 2 mM glutamine (Euroclone). Cells were incubated at 37°C in a humidified atmosphere of 5% CO_2_-95% air.

### Clinical specimens and fibroblast cultures

Breast cancer tissues and their relative normal counterparts (normal mammary skin samples) were obtained from patients who underwent surgery at the *Unità Operativa di Chirurgia Senologica*, *Facoltà di Medicina e Chirurgia “A. Gemelli”*, *Università Cattolica del Sacro Cuore* (Rome, Italy), according to the principles expressed in the Declaration of Helsinki. Donors gave written informed consent to participate in the study.

Breast cancer and normal mammary skin tissue samples were minced into small pieces (2–3 mm^3^) and attached onto 100 mm culture dishes. Primary normal mammary skin (NFs) and cancer-associated (CAFs) fibroblasts isolated from clinical specimens were maintained in DMEM (Euroclone) supplemented with 10% FBS (Life Technologies), antibiotics (100 IU/ml penicillin, 100 µg/ml streptomycin, Euroclone), 2 mM glutamine (Euroclone) and 1 mM sodium pyruvate (Euroclone). Cells were incubated at 37°C in a humidified atmosphere of 5% CO_2_-95% air.

The fibroblastic nature of the isolated cells was verified by microscopic determination of morphology portrait and immunocytochemical characterization using fibroblast- (anti-vimentin, V9 clone, Dako, Glostrup, Denmark); epithelial tumor cell- (anti-cytokeratin 18, DC 10 clone, Dako), activated fibroblast- (anti-α-smooth muscle actin, α-SMA, 1A4 clone, Dako), and endothelial cell- (anti-CD31, 89C2 clone, Cell Signaling Technology, Beverly, MA, USA) specific markers. NFs used for co-culture assays were immunoreactive for vimentin but negative for all the other markers. CAFs used for co-culture assays were immunoreactive for vimentin and α-SMA but negative for cytokeratin 18 and CD31. Co-culture experiments were carried out with fibroblasts (NFs and CAFs) of passage 2–3 to accumulate large amounts of cells for co-cultivation while avoiding cell aging effects.

### 3D cultures/co-cultures

For the immnunohistochemical/immunoflurescence analysis, primary fibroblasts (NFs or CAFs) were directly co-cultured with MCF-7 and MDA-MB-231 cells in a 3D model (nodules) as previously described [23]. Briefly, after detachment of cell monolayers with trypsin/EDTA, the cell density was arranged to 2.5×10^5^ cells/ml for each cell type. One ml of the cell suspension was distributed in 5-ml centrifugation tubes containing semi-solid medium (final concentration: 1×DMEM-0.5% agar-agar). Cells were spinned down onto the semi-solid medium by centrifugation at 800 g for 10 min at 12°C. Most of the supernatant liquid medium was removed and the tubes incubated overnight in an incubator at 37°C. The next day, the pellets were recovered with 200 µl of 1×DMEM by gentle aspiration and transferred on 1006-Falcon Petri dishes containing 2 ml of semi-solid medium. Medium was renewed 2 days later and then after 5 days.

For co-cultures, a 1∶1 proportion of each cell type was used. Homotypic cultures of only one cell type were used as controls.

After 4 or 6 days of culture, nodules were harvested for immunohistochemical or immunofluorescence analysis.

### 2D cultures/co-cultures

For the evaluation of vimentin expression, cell membrane fluidity and migration, conventional 2D adherent cultures were used. Briefly, confluent fibroblast and breast cancer cell cultures were trypsinized and 0.5×10^5^ cells of each cell type were plated together in 35 mm glass-bottom Petri dishes (ibidi GmbH, Martinsried, Germany) for confocal microscopy analysis in their standard medium, which was renewed after 3 days. After for 6 days, membrane fluidity or cell migration were evaluated as described below. Homotypic cultures of only one cell type (1×10^5^ cells) were used as controls.

### Immunohistochemistry

To verify the distribution of the two cell types in 3D co-cultures, and the effects of the heterotypic interactions on fibroblast activation and cell growth, immunohistochemical analysis of pankeratin, α-SMA and proliferation markers (Ki67 and proliferating cell nuclear antigen, PCNA) was performed on cryostat 10-µm-thick sections from 4- and 6-day frozen nodules. Nodules were previously fixed in 4% paraformaldehyde/phosphate-buffered saline (PBS), cryoprotected in 30% sucrose, and frozen in liquid nitrogen. Sections were collected on superfrost slides (Thermo Scientific, Fremont, CA, USA), dried for 15 min at room temperature (RT) and blocked with Superblock (UCS Diagnostic, Rome, Italy) for 10 min. Antigen retrieval was accomplished by microwave heating (500 W) in 10 mM sodium citrate buffer of pH 6.0 for 5 min. The sections were incubated with anti-cytokeratin (large spectrum, pankeratin, KL1 clone; UCS Diagnostic; 1∶100 dilution), anti-α-SMA (1A4 clone, Dako; 1∶60 dilution), anti-Ki67 (MIB-1 clone, Dako; 1∶100 dilution) and anti-PCNA (PC10 clone, Dako; 1∶150 dilution) 1 h at RT in PBS. Then, a secondary antibody conjugate (HRP polymer conjugate, broad spectrum, Invitrogen) was applied for 30 min at RT. The chromogenic reaction was developed with 3,3′-diaminobenzidine tetrahydrochloride (DAB) solution (Zymed Labs., South S. Francisco, CA, USA). The nuclei were lightly counterstained with Mayer's hematoxylin. Negative controls without primary antibodies were performed. For proliferation markers (Ki67 and PCNA), three sections from three different nodules for each culture condition were examined by two independent observers. The number of positive cells (on a total of at least 800 cells) was assessed in four randomized high-power fields (×400) for each section. All the labelled tumor nuclei, regardless of the staining intensity, were considered positive. A mean percentage for every sample was obtained and data were reported as mean±SD.

### Immunofluorescence and Confocal Microscopy Analysis

To assess the effect of tumor cell/fibroblast interaction on cell adhesion, immunofluorescence analysis of E- and N-cadherin was performed on cryostat 10 µm-thick frozen sections obtained as above described from 6-day nodule homotypic and heterotypic cultures. Sections were collected on superfrost slides (Thermo Scientific), dried for 15 min at RT and blocked with Superblock (UCS Diagnostic) for 10 min. Antigen retrieval was accomplished by microwave heating (500 W) in 10 mM sodium citrate buffer (pH 6.0) for 5 min. Mouse monoclonal primary antibodies anti-E-cadherin (4A2C7 clone, Invitrogen, Carlsbad, CA, USA; 1∶200 dilution) and anti-N-cadherin (GC-4 clone, Sigma-Aldrich, St. Louis, MO, USA; 1∶1000 dilution) were applied for 1 h at RT in PBS. Sections were then incubated with the secondary antibody (Cy3-conjugated AffiniPure Donkey Anti-Mouse IgG, 1∶200, Jackson ImmunoResearch Labs., West Grove, PA, USA) for 1 h at RT in PBS. Slides were mounted using 4′,6-diamino-2-phenylindole (DAPI) containing mounting medium (Vector Labs., Burlingame, CA, USA). Negative controls without primary antibodies were performed for all reactions. Images were collected by using an inverted confocal microscope (DMIRE2, Leica Microsystems, Wetzlar, Germany). They were obtained using a ×40 oil immersion objective (NA 1.4) equipped with a mode-locked Titanium-Sapphire laser (Chamaleon, Coherent, Santa Clara, CA) for DAPI excitation at 750 nm and with an Ar/ArKr laser for Cy3 excitation at 543 nm. Internal photon multiplier tubes collected eight bit unsigned images at a 400 Hz scan speed. Image background values (defined as intensities below 7% of the maximum intensity) were set to zero and colored black. Imaging was performed at RT.

Fluorescence Intensity values for each sample were quantified over multiple intensity images and ROIs and their mean±SD (n = 15 to 20) [24]–[27] determined and utilized for further statistical analysis (two-tailed Student's *t*-test).

### Western blot analysis

Cells of 6-day monolayer cultures were collected to be analyzed for verify the co-culturing effect on vimentin expression. Cells in mixed cultures were separated by magnetic cell sorting, using the EasySep™ Magnet (STEMCELL Technologies, Voden Medical Instruments SpA, Milan, Italy) according to the manufacturer's instructions. All cells were lysed in RIPA buffer [50 mM Tris-HCl (pH 7.7), 150 mM NaCl, 1% Triton X-100, 1% sodium deoxycholate, 0.1% SDS] freshly supplemented with phosphatase and protease inhibitors (100 µM Na_3_VO_4_, 0.3 mM phenylmethylsulfonylfluoride, 50 µg/ml leupeptin and 20 µg/ml aprotinin) on ice and the lysates clarified by centrifugation at 12,000 rpm for 10 min at 4°C. Samples containing 30 µg of total protein were resolved on a 10% SDS-PAGE and transferred onto Immobilon P membrane (Millipore, Bedford, MA, USA) which was incubated (1 h, at RT) with anti-vimentin antibody (clone V9, Dako, 1∶1000 dilution) in Tris buffered saline (TBS) containing 0.02% Tween 20 and 5% nonfat dried milk (blocking buffer). The blot was then overlaid with a HRP-labelled secondary antibody (Vector Labs.; 1∶2000 dilution) for 40 min in blocking buffer at RT. The protein bands were detected using an enhanced chemiluminescence system (ECL, Amersham, Buckinghamshire, UK) and visualised on Hyperfilm ECL (Amersham). Membranes were reprobed with an anti β-actin monoclonal antibody (clone AC15, Sigma-Aldrich; 1∶10,000 dilution) as an internal control for protein loading. The signals were quantified by densitometry (Chemi Doc Documentation System/Quantity One quantitation software, Bio-Rad).

### Laurdan two photon microscopy

To investigate the impact of tumour cell/fibroblast interactions on the respective membrane lipid packing density, homotypic or heterotypic cultures, maintained for 6 days in 35 mm glass-bottom Petri dishes, were labelled with 2-dimethylamino(6-lauroyl)naphthalene (Laurdan, Molecular Probes, Inc., Eugene, OR, USA), a ratiometric fluorescent dye highly sensitive to the presence and mobility of water molecules within the membrane bilayer, yielding information on membrane order and fluidity by a shift in its emission spectrum [28]. The probe was successfully applied to study membrane fluidity changes in living cells [29], [30]. Ratiometric probes allow the use of a ratio between two fluorescence intensities. This makes possible the correction of artifacts due to bleaching, changes in focus and variations in laser intensity [31]–[38]. The Laurdan stock solution concentration was 2 mM in dimethyl sulfoxide (Sigma-Aldrich), and it was renewed every three weeks. Laurdan labelling was performed directly in the culture media by adding the probe to the cells in culture at a final concentration of 2 µM. After 30–45 min of incubation in the dark at RT, cells were washed once with the complete culture medium and dishes mounted on an inverted confocal microscope (DMIRE2, Leica Microsystems) slide. Laurdan intensity images were obtained using a ×40 oil immersion objective (NA 1.4) under excitation at 800 nm with a mode-locked Titanium-Sapphire laser (Chamaleon, Coherent, Santa Clara, CA). Internal photon multiplier tubes collected eight bit unsigned images at a 400 Hz scan speed. Laurdan intensity images were recorded simultaneously with emission in the range of 400–460 nm and 470–530 nm and imaging was performed at RT.

#### Image analysis

Membrane fluidity can be measured in terms of ratio of emission intensities by using Generalized Polarization (GP) value, defined as
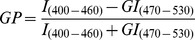
GP value was calculated for each pixel using the two Laurdan intensity images (I_(400–460)_ and I_(470–530)_) using the software Ratiometric image processor 2.0 [31]. The calibration factor G was obtained from the GP values of solutions of Laurdan in DMSO. G factor had ∼2% variation across the imaging area. GP images (as eight-bit unsigned images) were pseudocoloured in Image-J. Background values (defined as intensities below 7% of the maximum intensity) were set to zero and coloured black. GP histograms values were determined within multiple Regions of Interest (plasma membranes of single cells) for each sample, and their mean±SD (n = 15 to 20) determined and utilized for further statistical analysis (two-tailed Student's *t*-test). Line profiles and analysis of acquired images were performed with image J.

### Migration assay

To evaluate the reciprocal influences of tumour cells and fibroblasts on the respective migratory ability, confocal microscopy was performed in a temperature controlled room (T = 30°C) on cells (homotypic and heterotypic cultures) cultured for 6 days in 35 mm glass-bottom Petri dishes. In each experiment, the single cell type of interest (tumor cells or fibroblasts) has been labelled with the fluorescent probe CellTracker Green CMFDA (Invitrogen) that is retained in living cells through several generations and is not transferred to adjacent cells. Thus, the single cell movement was analyzed over a time period of 45 min. The time interval between consecutive frames was one min. Background noise was subtracted from each image. Cell tracking [39] was performed by using the ImageJ software plugin “ParticleTracker” [40]. On average 40 cells per experiment were tracked. Once the analysis region was defined by means of the feature point detection and tracking algorithm, each cell was numbered and its (*x(t_i_), y(t_i_)*) position coordinates measured for each time point *t_i_* (*i* = 1,….,N = 45). We used the ImageJ-plugin “Chemotaxis and migration tool” to analyze kinematic quantities of migration processes: the migration velocity, the Euclidean and accumulated distances and the directness [41]. The instantaneous migration velocity for each cell was calculated as 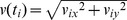 where *v_ix_* and *v_iy_* were calculated according to the expression:
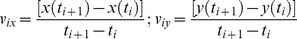
Where (*x(t_i+1_), y(t_i+1_)*, (*x(t_i_), y(t_i_)*)) are the positions of each cell at the time *t_i+1_* and *t_i_*. The mean migration velocity 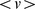 was calculated according to the expression:
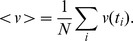
The Euclidian distance *d_E_* for each cell was calculated as the length of the straight line between the cell start point (*x(t_1_), y(t_1_)*) and the end point(*x(t_N_), y(t_N_)*):
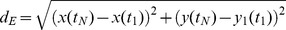
The accumulated distance *d_A_* for each cell is the result of the sum of all incremental movements measured in between consecutive images:
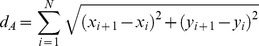
The directness, which represents a measure of the cell's tendency to travel in a straight line, is calculated as the ratio of the Euclidian distance and the accumulated distance.
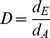
A directness of *D* = 1 indicates a straight-line migration from the start point to the end point.

### Statistical analysis

At least three independent experiments were performed for each protocol. The two-tailed Student's *t*-test was used to compare means of two different groups; ANOVA and the Tukey's multiple comparison test were used to study differences of means of multiple samples. The level of significance was set at p<0.05.

## Results

### Characterization of isolated fibroblasts

**Figure 1:**
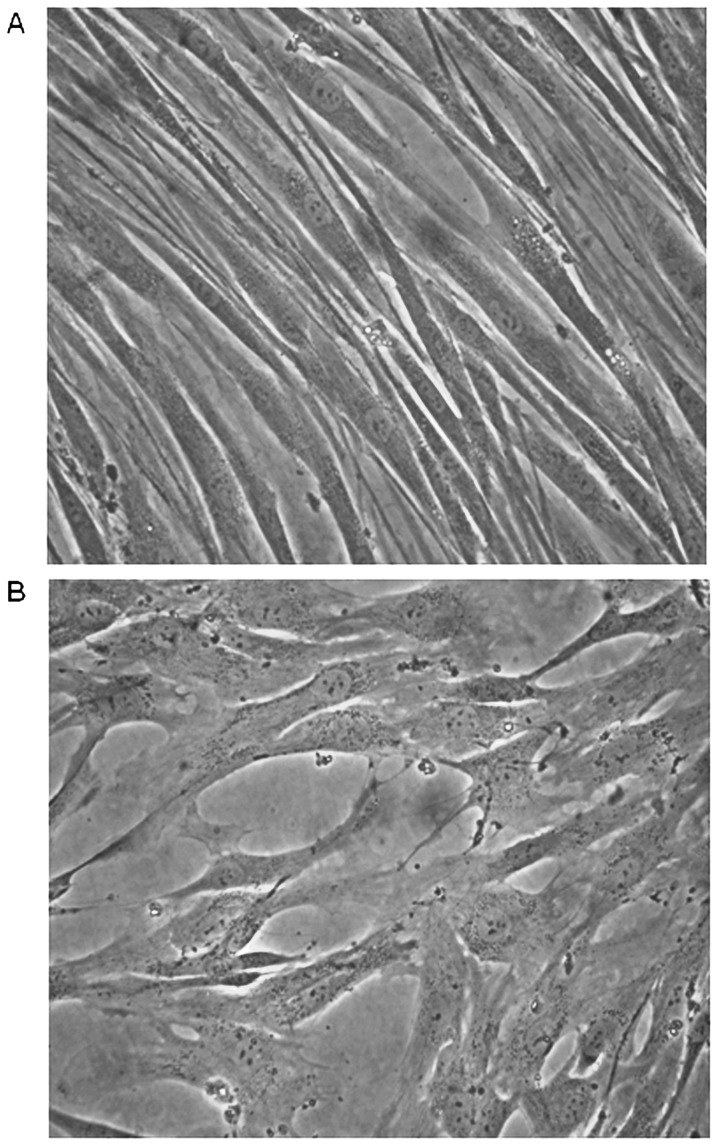
Morphology of isolated normal and tumor-derived fibroblasts. Light microscopic view showing the different morphology of fibroblasts isolated from human healthy mammary skin (A) or human breast cancer (B) samples after 6 days of culture. Original magnification, ×200.

Immunocytochemically characterized populations of the isolated mammary fibroblasts (normal dermal and cancer-associated) stained uniformly for vimentin, but resulted negative for cytokeratin 18 and CD31, confirming the stromal origin of cells and the absence of contaminating epithelial or endothelial elements. As reported in literature [42], approximately 10–12% of the CAFs were positive for α-SMA, while among the NF population only very few elements showed α-SMA immunoreactivity, probably induced by the culture condition (i.e. 2D cultures) (data not shown). Morphologically, NFs in culture showed the typical spindle-shape fibroblast-like appearance (Figure 1A), while CAFs exhibited a more irregular and flattened morphology (Figure 1B).

### Spatial distribution of tumor/fibroblast cells in 3D co-cultures

**Figure 2:**
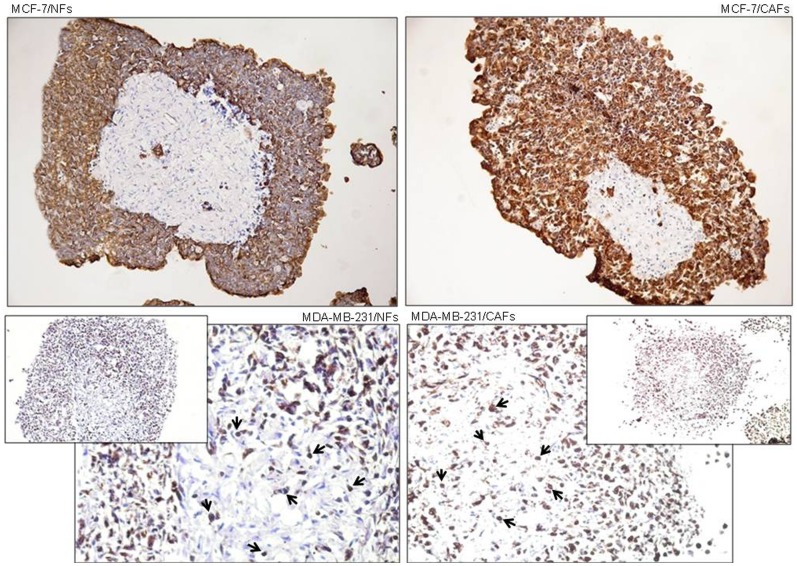
Spatial distribution of breast tumor cells and fibroblasts in nodule co-cultures. Sections (10 µm) of paraformaldehyde-fixed frozen nodule co-cultures of either MCF-7 or MDA-MB-231 breast tumor cells with fibroblasts (NFs or CAFs) at day 6 of culture are shown. Sections were stained immunohistochemically using an anti-cytokeratin (large spectrum, pankeratin) monoclonal antibody to identify the reciprocal location of the two cell types (tumor cells and fibroblasts) in the nodule co-cultures. The more invasive and less differentiated MDA-MB-231 cells showed a looser association as compared to MCF-7/fibroblast co-cultures, with various cells infiltrating the fibroblast nodule core (arrows). DAB for detection; hematoxylin counterstaining; original magnification for upper panels (MCF-7/NFs, MCF-7/CAFs) and lower insets, ×100; original magnification for lower panels (MDA-MB-231/NFs and MDA-MB-231/CAFs), ×200.

Representative cytokeratin-stained sections from tumor/fibroblast nodule co-cultures (MCF-7/NFs, MCF-7/CAFs, MDA-MB-231/NFs, MDA-MB-231/CAFs) at day 6 are shown in Figure 2, indicating the spatial distribution of tumor cells (stained with the epithelial marker cytokeratin) and fibroblasts in this culture model. As expected, in MCF-7/fibroblast cell nodules fibroblasts were concentrated in the central zone leaving a strip of almost pure cancer cells at the periphery of the nodules. In the MDA-MB-231/fibroblast cell co-cultures, a more diffuse distribution of the two cell types was observed. In contrast to the less invasive MCF-7 cells, the highly invasive MDA-MB-231 cells extensively infiltrated the fibroblast core, particularly in the presence of CAFs.

Similar cell distributions were observed at day 4 (data not shown).

### Highly invasive and metastatic breast tumor cells induced fibroblast activation

**Figure 3:**
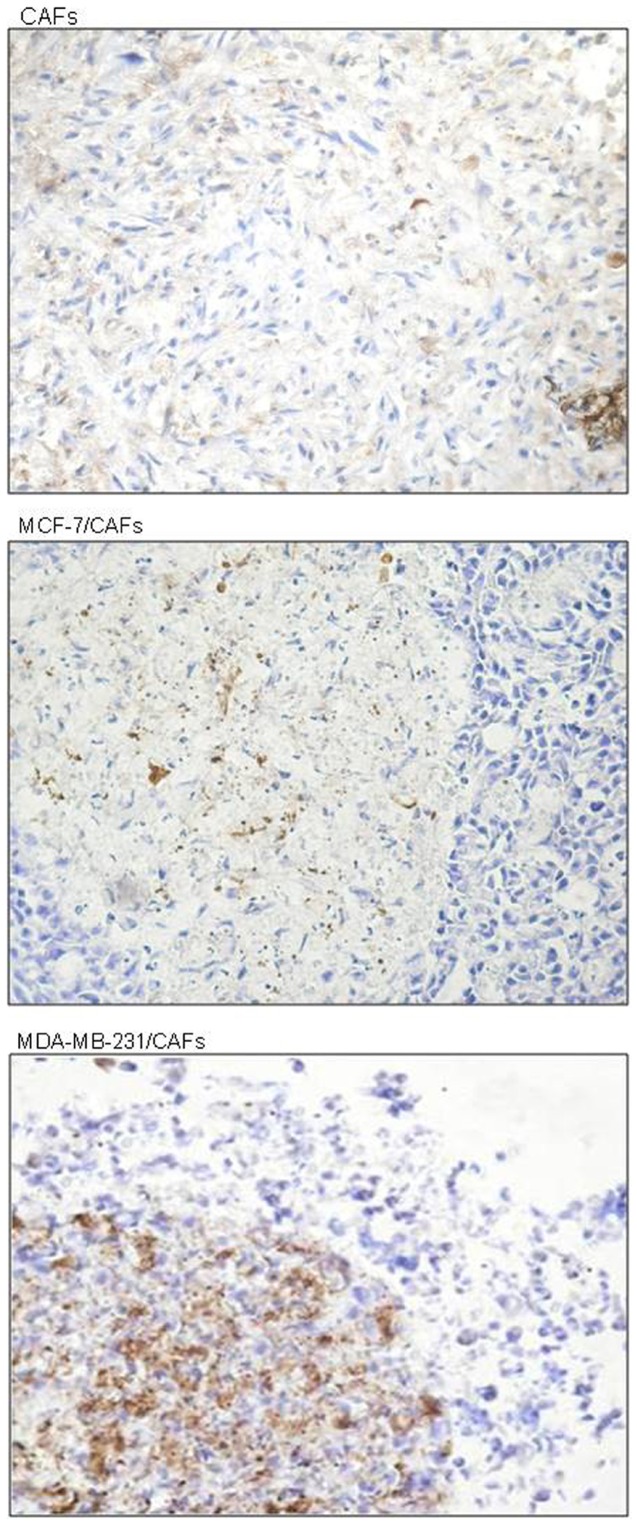
Breast tumor cell induction of α-smooth muscle actin (α-SMA) in tumor stroma-derived fibroblasts. Sections (10 µm) of paraformaldehyde-fixed frozen nodule culture (CAFs) and co-cultures (MCF-7 or MDA-MB-231 breast tumor cells with CAFs) at day 6 of culture are shown. Sections were stained immunohistochemically using an anti-α-SMA monoclonal antibody to verify the effect of breast cancer cells on fibroblast-to-myofibroblast transdifferentiation. α-SMA expression was induced in the tumor-associated fibroblast population as a result of the interaction with the more aggressive and less differentiated MDA-MB-231 cells, demonstrating the ability of these cells to promote stroma activation. DAB for detection; hematoxylin counterstaining; original magnification, ×200.

The immunocytochemical analysis for α-SMA, demonstrated that the highly invasive and metastatic MDA-MB-231 cells, when co-cultured with CAFs for 6 days, induced a sharp increase in the amount of α-SMA-positive stromal elements, indicating the acquisition of a myofibroblast-like phenotype, typical of the breast tumor stroma (Figure 3). The poorly invasive and low metastasizing MCF-7 breast cancer cells were not able to influence α-SMA expression of tumor-associated fibroblast population (Figure 3). Neither the highly nor the poorly invasive tumor cells were able to induce activation of normal fibroblasts (data not shown). The same results were obtained after 4 days of co-culturing (data not shown).

### Cancer-associated fibroblasts exerted a mitogenic effect on breast tumor cells

**Figure 4:**
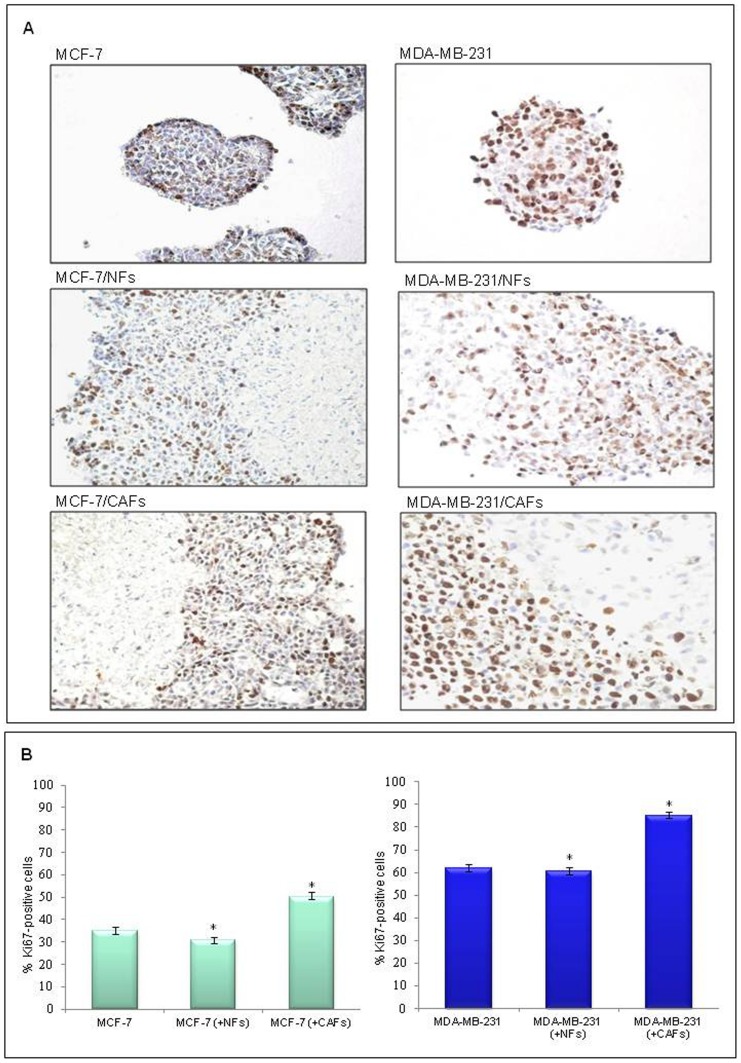
Cancer-associated fibroblasts but not their normal counterpart exerted mitogenic effect on breast cancer cells. (A) Sections (10 µm) of paraformaldehyde-fixed frozen nodule cultures (MCF-7 or MDA-MB-231 breast tumor cells) and co-cultures (MCF-7 or MDA-MB-231 cells with NFs or CAFs) at day 6 of culture are shown. Sections were stained immunohistochemically using an anti-Ki-67 monoclonal antibody (MIB-1 clone) to verify the reciprocal effect of the two cell types on their respective growth rate. The MCF-7 cell/CAF mixed nodules showed a higher number of Ki67-positive tumor cells with respect to that observed in MCF-7 cell/NF or MCF-7 cell nodules. Co-cultures of MDA-MB-231 cells and fibroblasts showed the same behavior as observed for MCF-7 cell/fibroblast nodule co-cultures.. DAB for detection; hematoxylin counterstaining; original magnification, ×200. (B) Quantitative representation of the immunohistochemical data. Three sections, obtained from 3 different nodules, were analyzed by two independent observers. The number of Ki67-positive cells (on a total of at least 800 cells) was assessed in four randomized high-power fields (×400) for each section. A mean percentage for every sample was obtained and data were reported as mean±SD (error bars). Statistical significance was determined using ANOVA and the Tukey's multiple comparison test, *p<0.001.

To investigate the reciprocal effects of cancer cells and fibroblasts on their respective cell growth, immunohistochemical analysis of proliferation markers (Ki67 and PCNA) was performed on paraformaldehyde-fixed frozen nodule sections. As shown in Figure 4A, Ki-67 mainly displayed a uniform nuclear staining. A weak cytoplasmic labelling was rarely observed but it did not interfere with the identification of positive nuclei. As expected, the percentage of Ki-67-positive cells was significantly higher in breast tumor cells co-cultured for 6 days with CAFs compared with control MCF-7 or MDA-MB-231 cell homotypic cultures. In fact, the MCF-7 cell/CAF mixed nodules showed a significantly larger proportion (50%; p<0.001) of Ki67-positive tumor cells compared to MCF-7 cells cultured alone (34.8%) or in the presence of NFs (30.6%) (Figure 4A and 4B). The same CAF-triggered mitogenic effects were observed in MDA-MB-231 cells as the percentage of Ki67-positive tumor cells was higher (85%, p<0.001) compared to that of either MDA-MB-231 cell cultures or MDA-MB-231 cell/NF mixed nodules (61.7% and 60.3%, respectively) (Figure 4A and 4B). Mammary cancer cells seemed to have no influence on the growth of both kinds of fibroblasts. Similar results were obtained on 4-day co-cultures and with PCNA, even though a more heterogeneous and less intense nuclear staining was observed (data not shown).

### Cancer-associated and normal fibroblasts influenced E- and N-cadherin expression in breast tumor cells

**Figure 5:**
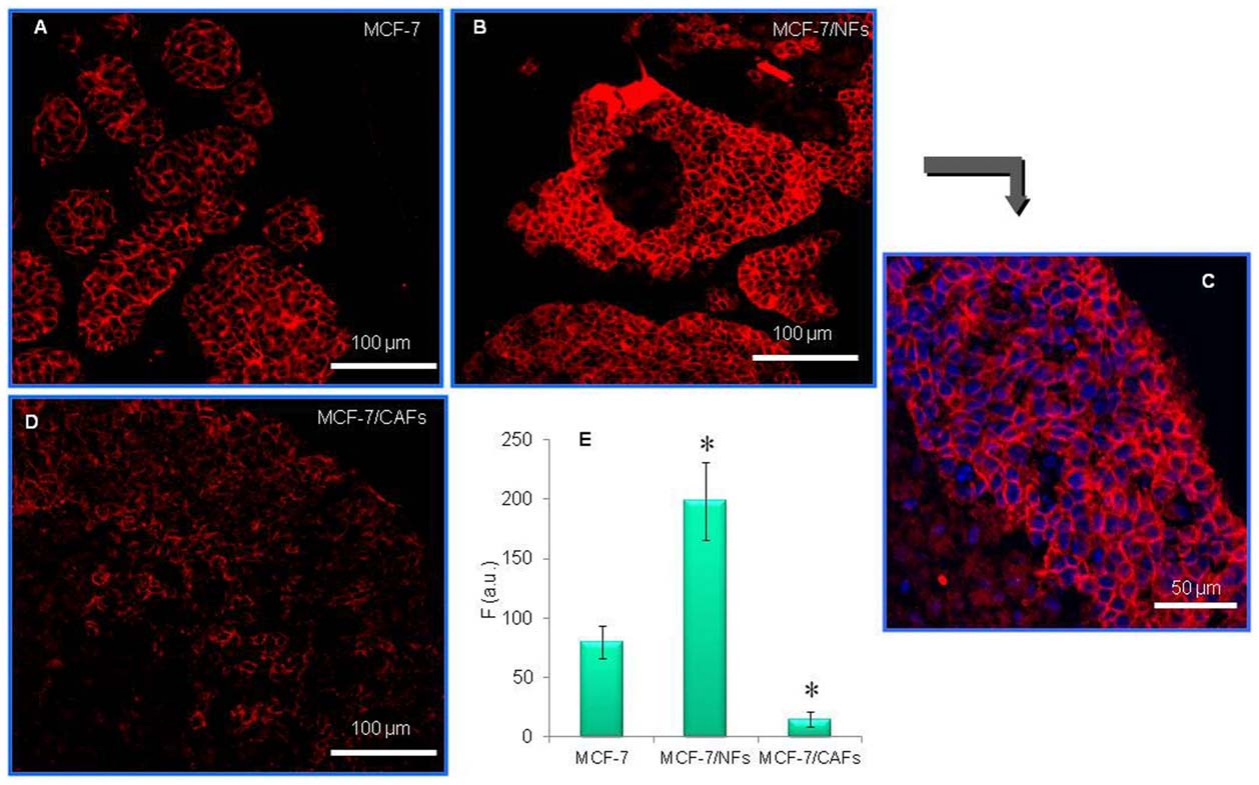
Cancer-associated fibroblasts and their normal counterpart affected E-cadherin expression in breast cancer cells. Frozen nodule sections from MCF-7 cell homotypic cultures (A) or MCF-7 cell/fibroblast mixed cultures (B and D) were processed for E-cadherin immunostaining (Cy3: red). DAPI was used for nuclear counterstaining (blue) (C). Confocal images show the up-regulation of E-cadherin in MCF-7 cell population co-cultured with NFs (B) as compared to the MCF-7 homotypic culture (A). On the contrary, interaction with CAFs promoted a strong reduction in E-cadherin expression (D). A representative experiment of three is shown. (E) *F (a.u.)*, fluorescence intensity (in arbitrary units). Data are mean±SD (error bars) of three independent experiments. Statistical significance was determined using Student's t-test, *p<0.05.

To test the reciprocal effects of cancer cells and fibroblasts on the respective adhesiveness, a confocal immunofluorescence study of E- and N-cadherin expression was performed on nodule sections. The basal level of E-cadherin expression in MCF-7 cells is shown (Figure 5A) and quantified (Figure 5E). The analysis demonstrated the ability of NFs to induce E-cadherin expression in MCF-7 cells after 6 days of co-culture, when a great increase in signal intensity (∼2.5-fold, compared with MCF-7 cell homotypic cultures, Figure 5B, 5C and 5E) was observed. On the other hand, a CAF-triggered down-regulation of the adhesion molecule (∼80%, compared with MCF-7 cell homotypic cultures) was seen (Figure 5D and 5E). An increase in N-cadherin level was observed in CAFs, as the result of a 6-day long interaction with both kinds of epithelial cancer cells (up to ∼180%, compared with CAF homotypic cultures). CAFs, in turn, promoted the up-regulation of N-cadherin in MDA-MB-231 cells (∼60%, compared with MDA-MB-231 cell homotypic cultures). CAF-induced N-cadherin expression was found in the N-cadherin-negative MCF-7 cells. NFs were not able to influence N-cadherin expression in cancer cells, neither were they affected by tumor cells (data not shown).

For all the cell types similar results were obtained with 4-day co-cultures (data not shown).

### Cancer-associated fibroblasts induced vimentin expression in breast tumor cells

**Figure 6:**
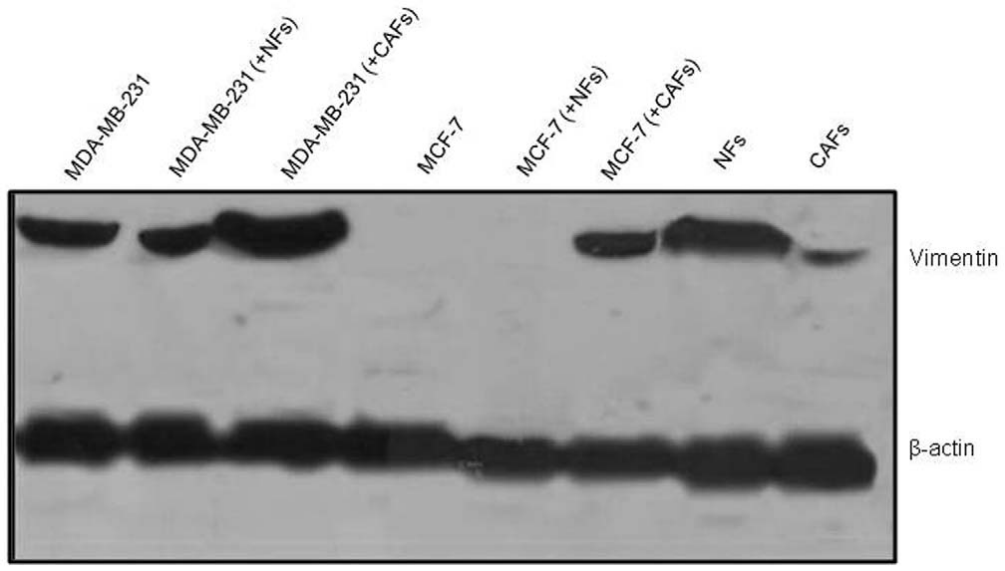
CAFs induced EMT in breast tumor cells. Western blot analysis revealed that the vimentin protein expression was clearly induced in the low-invasive MCF7 cell line co-cultured with CAFs. Vimentin levels were increased in the highly invasive MDA-MB-231 cells co-cultured with CAFs as compared with the control (MDA-MB-231 cells cultured alone). No effect on vimentin expression was found in breast tumor cells co-cultured with NFs.

As shown in Figure 6, the expression of the mesenchymal marker vimentin was clearly induced in the relatively well-differentiated breast cancer cells (MCF-7) co-cultured with CAFs. An up-regulation of the protein was observed in the poorly differentiated, mesenchymal-like MDA-MB-231 cells. NFs did not affect the expression of the marker in cancer cells.

### Breast tumor cells and fibroblasts reciprocally influenced cell membrane fluidity

**Figure 7:**
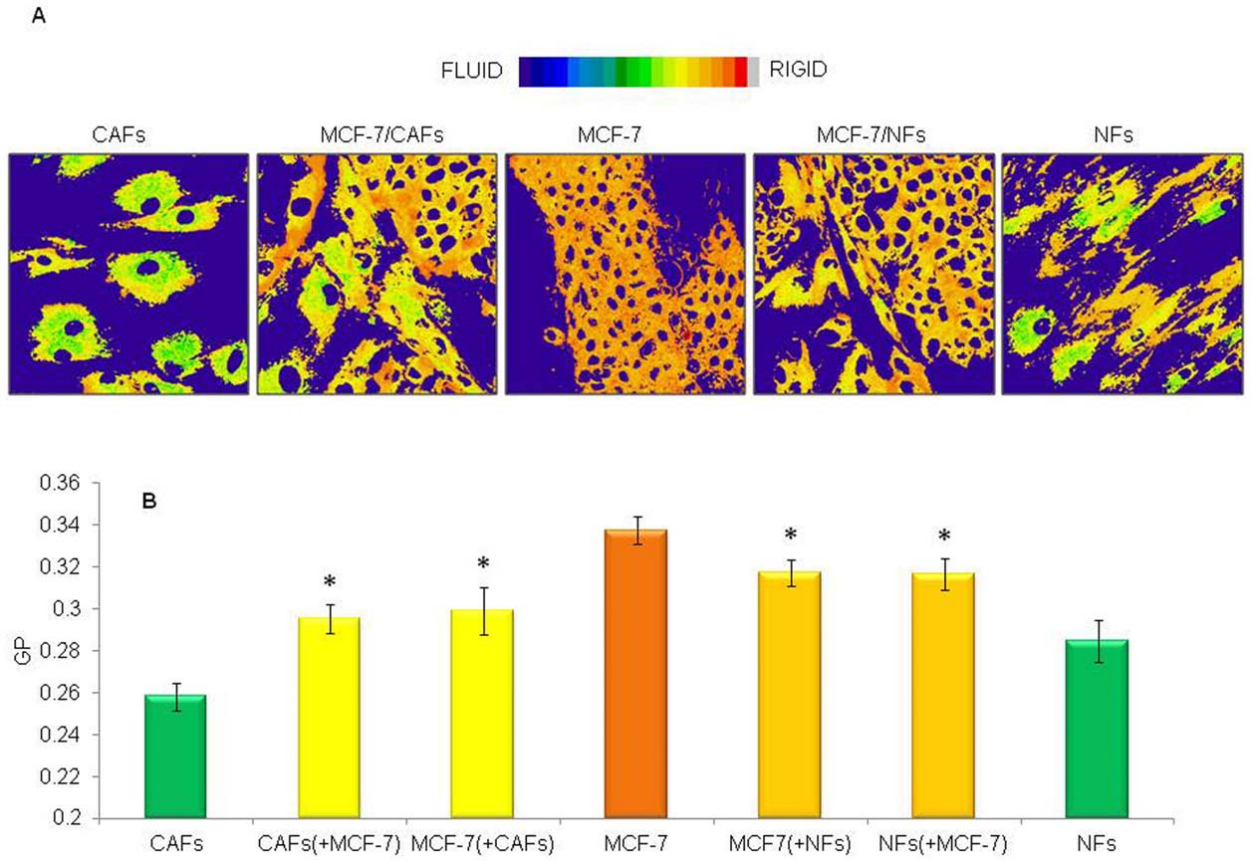
Low invasive breast cancer cells and fibroblasts reciprocally influenced their plasma membrane fluidity. Pseudocoloured GP images of living tumor (MCF-7) and fibroblast (NFs or CAFs) cells (A), and reciprocal effects on membrane fluidity (B) are shown. MCF-7 cells were cultured for 6 days, alone or in presence of NFs or CAFs, in 35 mm glass-bottom Petri dishes and labelled with Laurdan (2 µM). Tumor cell/fibroblast interaction determined an enhancement of cancer cell membrane fluidity. On the other hand, MCF-7 cells promoted an increase in fibroblast membrane packing density. Data are mean±SD (error bars) of three independent experiments. Statistical significance was determined using Student's t-test, *p<0.05 vs respective homotypic culture.

**Figure 8:**
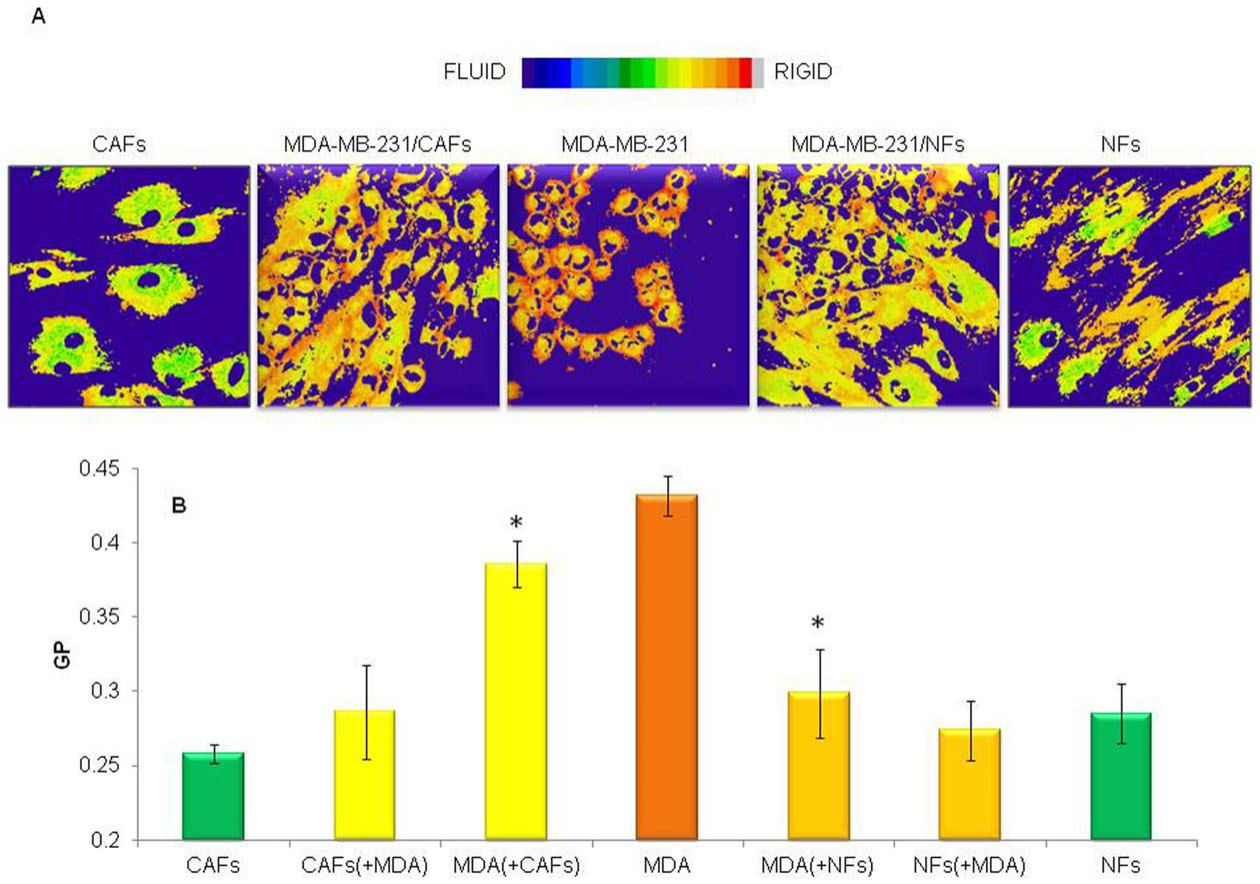
Highly invasive and metastatic breast cancer cells underwent a plasma membrane fluidity increase when co-cultured with normal or cancer-associated fibroblasts. Pseudocoloured GP images of living tumor (MDA-MB-231) and fibroblast (NFs or CAFs) cells (A), and reciprocal effects on plasma membrane fluidity (B) are shown. MDA-MB-231 cells were cultured for 6 days, alone or in presence of NFs or CAFs, in 35 mm glass-bottom Petri dishes and labelled with Laurdan (2 µM). Tumor cell/fibroblast interaction determined an enhancement of cancer cell membrane fluidity, while MDA-MB-231 cells did not affect fibroblast membrane packing density. Data are mean±SD (error bars) of three independent experiments. Statistical significance was determined using Student's t-test, *p<0.05 vs respective homotypic culture.

The impact of fibroblast/tumour cell interplay on the respective plasma membrane lipid packing density was studied by means of a widely used membrane fluorescent probe (Laurdan), an amphiphilic molecule whose fluorescence emission is highly sensitive to the microenvironment polarity and viscosity. As a result of a 6-day interaction between tumor cells and fibroblasts in 2D co-cultures, a significant enhancement of both MCF-7 (Figure 7A and 7B) and MDA-MB-231 (Figure 8A and 8B) cell membrane fluidity, demonstrated by reduction in GP values, was observed in the GP images and quantified within multiple Regions of Interest (plasma membranes of single cells) for each sample. On the other hand, the poorly invasive MCF-7 cells promoted an increase in the fibroblast membrane packing density (Figure 7A and 7B). After 6 days of co-culture, the GP values of MCF-7 cells and CAFs became nearly the same (GP∼0.30). Interestingly, this value corresponds to the arithmetic mean of the GP values of MCF-7 cells alone (GP∼0.34) and CAFs alone (GP∼0.26). As a result of MCF-7/NF interaction, the same phenomenon occurred (Figure 7A and 7B): the GP value of MCF-7 cells and NFs is again the same (GP∼0.32) and corresponds to the arithmetic mean of the GP values of control MCF-7 homotypic cultures (GP∼0.34) and control NF homotypic cultures (GP∼0.28).

On the contrary, the highly invasive and metastatic MDA-MB-231cells left unchanged the fibroblast membrane packing density (Figure 8A and 8B).

### Cancer-associated and normal fibroblasts influenced migration velocity and directness of breast tumor cells

**Figure 9:**
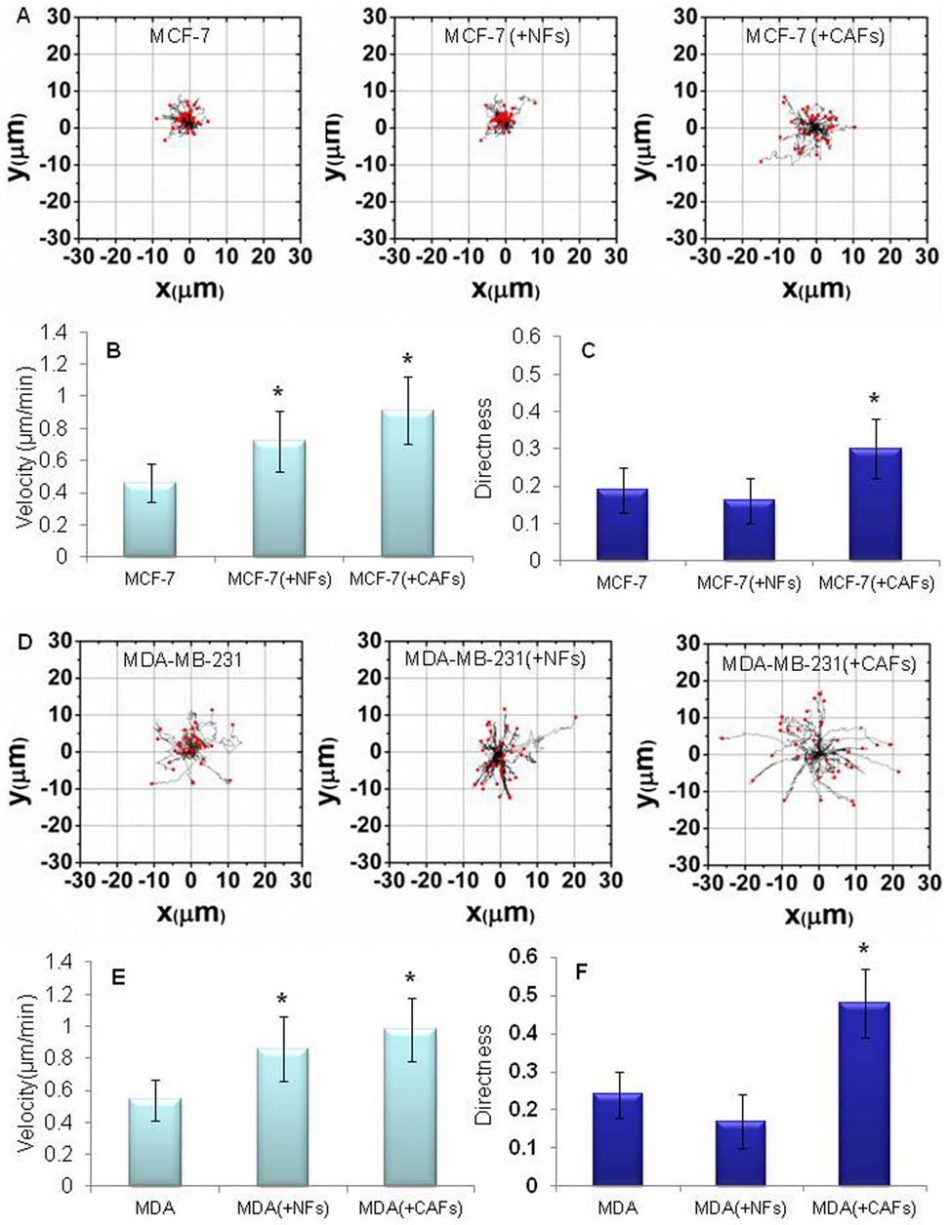
Breast tumor cells displayed increased migration velocity and directness in vitro due to their interaction with fibroblasts. Representative trajectory plots of MCF-7 (A) or MDA-MB-231 cells (D) evaluated over a time period of 45 min. Cells were cultured for 6 days, alone or in presence of NFs or CAFs, in 35 mm glass-bottom Petri dishes and labelled with the CellTracker Green CMFDA. The starting point of each cell trajectory is plotted at the center of the graph. A significant increment of migration velocity (in µm/min) of MCF-7 (B) or MDA-MB-231 (E) cells was induced by the interaction with both CAFs and NFs. A marked enhancement of the tumor cell migration directness was promoted by CAFs in MCF-7 (A, D) or MDA-MB-231 (C, F) cells. Data are mean±SD (error bars) of three independent experiments. Statistical significance was determined using Student's t-test, *p<0.05.

To test whether fibroblast/cancer cell interactions could influence specific aspects of the cell migratory behaviour, such as velocity and directness, we used single cell tracking of living cells and time-lapse confocal microscopy. We obtained representative trajectory plots of MCF-7 (Figure 9A) or MDA-MB-231 cells (Figure 9D) evaluated over a time period of 45 min. As shown in Figure 9B, migration velocity was significantly increased in MCF-7 cells co-cultured with NFs (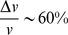) or CAFs (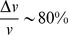), compared with control MCF-7 homotypic cultures. CAFs seemed to be more efficacious than NFs in promoting this effect which was also associated with an increase in directness of MCF-7 cell movements (Figure 9A, 9B and 9C). The same effects were obtained when the more invasive MDA-MB-231 cells were co-cultured with fibroblasts (Figure 9D, 9E and 9F). On the other hand, the presence of breast cancer cells did not affect the migratory ability of either NFs or CAFs (data not shown).

## Discussion

The influence of the surrounding stromal environment on breast cancer cell behaviour has been widely recognized but the parameters involved and their relationship are still far from being fully elucidated. Defining the differences between the so called reactive stroma of breast tumors and the normal stroma in terms of their influence on tumor cell migratory/invasive properties is therefore of great interest to deepen the knowledge of the mechanisms responsible of cancer progression and metastasis. In this paper, we used conventional 2D monolayer or 3D cultures to investigate the reciprocal influence occurring between breast cancer cells with different degree of differentiation/invasiveness and fibroblasts derived from breast cancer stroma (CAFs) or their normal counterpart (NFs) on some structural and functional features strongly correlated with the invasive/metastasizing phenotype. We found an overall migration/invasion-promoting effect of CAFs on both low and highly invasive mammary cancer cells, as demonstrated by their ability to increase tumor cell growth, promote epithelial-mesenchymal transition, reduction of cell adhesion, enhancement of membrane fluidity, and migration velocity and directness. These effects were not found or were less intense when cancer cells were grown with NFs under the same experimental conditions. Notably, NFs seem to favour the maintenance of the normal tissue architecture strongly promoting E-cadherin expression. Based on these findings, we conclude that in these experimental models CAFs are able to interact with cancer cells and modulate their migratory behaviour with a greater efficiency, if compared to NFs.

In regard to the effect of tumor cell/stroma interaction on cell growth, cohabiting with CAFs induced a clear mitogenic response in both low and highly invasive breast cancer cells, in agreement with previous studies [43]. The MCF-7 cell/CAF and MDA-MB-231/CAF mixed nodules showed a significantly larger proportion of Ki67- or PCNA-positive tumor cells with respect to that observed in the homotypic cultures as well as in the mixed nodule sections of tumor cells and NFs. Even though mammary cancer cells seemed to have no influence on the fibroblast growth, the more invasive and metastatic ones promoted fibroblast-to-myofibroblast transdifferentiation. This effect was revealed by the sharp increase in the percentage of α-SMA-positive stromal elements found in the CAF population co-cultured with MDA-MB-231 cells which indicates the acquisition of a myofibroblast-like phenotype, typical of the breast tumor stroma activation [44]. Consistent with data by other authors [42], these findings suggest that α-SMA expression in stromal fibroblast (fibroblast activation in tumor stroma) is influenced by the interacting cancer cell type.

Epithelial-to-mesenchymal transition (EMT) characterizes progression of many types of carcinomas. Changes in intercellular adhesions mediate this switch and dictate the cell receptivity towards signals from the extracellular milieu [45]. In particular, down-regulation or loss of the epithelial adhesion molecule E-cadherin represents a key step in the acquisition of the invasive phenotype for many tumors. This event is frequently associated with the up-regulation of the mesenchymal adhesion protein N-cadherin, strongly influencing cell motility. Indeed, N-cadherin was shown to be expressed in the most invasive and dedifferentiated breast cancer cell lines and its exogenous expression in malignant cells induces a motile scattered phenotype also enabling circulating tumor cells to associate with stromal and endothelial cells at distant sites [46]. A significant result of the present study is NFs' ability to induce a strong E-cadherin enhancement in MCF-7 cells, demonstrated by confocal immunofluorescence microscopy on frozen nodule sections. This result disagrees with recent findings by Gao et al. [47], who described a NF-triggered down-regulation of E-cadherin in the same tumor cells by Western blot analysis. On the other hand, consistent with data from these authors [47], we observed a CAF-triggered reduction of this adhesion molecule. For the first time, in our experience an increase in N-cadherin level was observed in CAFs, but not in NFs, as the result of the interaction with both low and highly invasive breast cancer cells. CAFs, in turn, promoted the up-regulation of N-cadherin in MDA-MB-231 cells and, as previously reported [47], its *de novo* expression in MCF-7 cells. The EMT-promoting activity of CAFs, demonstrated by their ability to trigger “cadherin switching”, was corroborated by the induction of the mesenchymal marker vimentin in the well differentiated MCF-7 cells and its up-regulation in the highly dedifferentiated MDA-MB-231 cells. It is conceivable that these CAF-promoted molecular changes of the tumor cell phenotype could have relevant implications in cancer progression and metastasis, not only promoting invasiveness, but also enabling interactions between tumor cells and the N-cadherin-expressing elements of the surrounding microenvironment, also in metastatic niches.

Changes in the motional freedom of lipids and protein molecules in the cellular membrane represent a critical feature of pathological cells, influencing several aspects of their behaviour such as the response to chemotherapeutic agents [48], [49], availability or activation of membrane receptors [50], [51], migratory, invasive and/or metastatic potential [52], [53]. Even though no univocal trend is evident regarding the direction of alterations in tumor cell membrane fluidity [54], in most tumors malignant cell membranes have been found to possess higher fluidity than those of their healthy counterparts. It has also been shown that such structural change significantly correlates with an increased migratory/invasive aptitude and malignant potential of tumor cells as well as with a poor prognosis [52], [53], [55], [56]. Therefore, in the present study, two-photon microscopy imaging of cells labelled with the membrane fluorescent probe Laurdan was used to investigate whether fluidity changes in plasma membranes could occur as a result of tumor cell/fibroblast co-culturing. For the first time, we showed that such heterotypic interaction determined a significant enhancement of cancer cell membrane fluidity. On the other hand, the more differentiated and poorly invasive MCF-7 cells promoted an increase in fibroblast membrane packing density whose significance remains to be elucidated.

Cell adhesion, changes in membrane phase properties and migration are complex and inter-dependent cellular processes, whose alterations characterize EMT and lead to the acquisition of a more motile phenotype by cancer cells, allowing for tumour invasion and dissemination.

Hence, we finally studied the reciprocal influences of stromal and cancer cells on their respective migratory ability. The interaction of both poorly and highly invasive mammary cancer cells with NFs or CAFs determined a definite increment in tumor cell migration velocity. As expected, this migration-promoting activity was particularly noticeable for CAFs which were able to induce even a marked enhancement of the migration directness of breast tumor cells. On the other hand, no influence of breast cancer cells on the fibroblast migratory ability was detected. To the best of our knowledge, these findings represent the first evidence of the ability of fibroblasts residing in breast tumor stroma to promote both speed and directness of cancer cell migration. In addition, they support results obtained from membrane fluidity analysis.

Directional persistence, indicating the existence of a preferential cell track for migration, is an important component of cell motility controlled by multiple factors which include topography of the extracellular environment as well as intrinsic cell properties [57], [58]. It seems conceivable that stroma-induced alterations in specific tumor cell processes, such as cytoskeletal organization, integrin-mediated cell/substratum adhesion or intracellular force creation, have a part in triggering such effect. Future efforts will need to elucidate the CAF-mediated processes that drive tumor cells to migrate on preferential tracks.

In summary, our study provides a further insight into the mechanisms involved in breast cancer progression, revealing novel aspects of the reciprocal interactions occurring between malignant cells and the neighbouring fibroblast population. In particular, an overall progression- and invasion-promoting effect of CAFs on both well- and poorly-differentiated mammary cancer cells was expressed as their ability in inducing structural and functional changes favouring the acquisition of a motile and invasive phenotype. Moreover, the tumor cell-triggered enhancement in N-cadherin and α-SMA levels of CAFs, together with MCF-7 cell-induced rigidification of fibroblast membrane, seems to reflect the ability of breast cancer cells to induce changes in the stromal cell microenvironment, whose significance deserves to be deepened. Defining the reciprocal influence of CAFs and mammary cancer cells on specific cell properties which promote tumor invasion and shedding light on the molecular mechanisms that subtend these effects may help in identifying new key regulators of breast tumor progression.
